# Identification of Pathogenic Bacteria from Public Libraries via Proteomics Analysis

**DOI:** 10.3390/ijerph16060912

**Published:** 2019-03-14

**Authors:** Ryan Hyunjae Jung, Minzae Kim, Bhoomi Bhatt, Jong Min Choi, Jung H. Roh

**Affiliations:** 1 Carnegie Vanguard High School, Houston, TX 77019, USA; Ryan.Jung@bcm.edu; 2 Amador Valley High School, Pleasanton, CA 94566, USA; theminzae@gmail.com; 3 Verna & Marrs McLean Department of Biochemistry and Molecular Biology, Baylor College of Medicine, Houston, TX 77030, USA; Bhoomi.Bhatt@bcm.edu; 4 Advanced Technology Core, Baylor College of Medicine, Houston, TX 77030, USA; 5 Department of Microbiology and Molecular Genetics, McGovern Medical School, The University of Texas Health Science Center at Houston, Houston, TX 77030, USA

**Keywords:** pathogenic bacteria, proteomics analysis, library books, environmental risk

## Abstract

Hazardous organisms may thrive on surfaces that are often exposed to human contact, including children’s library books. In this study, swab samples were taken from 42 children’s books collected from four public libraries in Texas and California. Samples were then cultivated in brain–heart infusion (BHI) medium and then in Luria broth (LB) medium containing either ampicillin or kanamycin. All 42 samples (100%) were positive for bacterial growth in normal BHI medium. Furthermore, 35 samples (83.3%) and 20 samples (47.6%) in total were positive in LB medium containing ampicillin or kanamycin, respectively. Bacterial populations were then identified in samples using an Orbitrap Fusion™ Tribrid ™ mass spectrometer, a state-of-the-art proteomic analysis tool. Identified bacterial species grown in ampicillin included *Bacillus*, *Acinetobacter*, *Pseudomonas*, *Staphylococcus*, *Enterobacter*, *Klebsiella*, *Serratia*, *Streptococcus*, *Escherichia*, *Salmonella*, and *Enterococcus*. In contrast, identified bacteria grown in kanamycin included *Staphylococcus, Streptococcus, Enterococcus*, and *Bacillus*. The presences of pathogenic bacteria species were also confirmed. The results of this study warrant follow up studies to assess the potential health risks of identified pathogens. This study demonstrates the utility of proteomics in identifying environmental pathogenic bacteria for specific public health risk evaluations.

## 1. Introduction

Children’s library books may serve as a vector of contagious organisms as they are circulated throughout communities without established sterilization procedures. In 1985, concern about disease transmission through libraries were raised by McClary in his article “Beware the Deadly Books” [1]. In 1994, Brook et al. published that *Staphylococcus epidermidis* was recovered from four out of 15 public library books [2]. Recently, Rafiei et al. demonstrated that 20.8% of returned books from the Al-Zahra Hospital Library and the Library of Sciences Faculty of Isfahan University were culture-positive [3]. Identified bacteria included *Enterobacteriacease* and coagulase-negative *Staphylococcus*. Gamlale et al. reported that, while airborne fungi are found throughout the city of São Paulo, Brazil, they are present in higher concentrations in libraries, subsequently resulting in asthmatic or rhinitis symptoms in 49% of 314 interviewed librarians in a follow up study [4]. Currently, in the United States there are two reports demonstrating the presence of bacteria in library books [5,6]. However, these reports originate from university course assignments or high school science competitions; thus, full description of methods and results are not available as scientific literature. In addition, the samples of these reports consist of university library books and chapter books, rather than children’s books. Identification of organisms in children’s books in the United States has not been illustrated to our knowledge. Furthermore, organisms that children carry can vary from those of adults [7].

Identification of bacteria species using mass spectrometry-based proteomics has over 40 years of history. In 1970, pyrolysis–gas chromatography mass spectrometry was used to identify two microorganisms, *Micrococcus luteus* and *Bacillus subtilis* var. *niger* [8]. Since then, over 200 papers have been published per year for bacteria identification using the mass spectrometer. Advances in mass spectrometry techniques and instrument development have resulted in improved detection of bacteria species; however, most studies utilize matrix-assisted laser desorption/ionization—time of flight mass spectrometry (MALDI-TOF) [9,10,11,12,13,14,15,16,17,18,19]. Recently, in 2013 the U.S. Food and Drug Administration (FDA) approved two matrix-assisted laser desorption-time of flight-mass spectrometry (MALDI-TOF-MS)-based platforms for bacteria identification [20]. Though MALDI-TOF-MS-based identification is a well-established technique, it can only identify bacteria at the genus level, not at the species or subspecies level [20]. Liquid Chromatography-Electrospray Ionization-Tandem Mass Spectrometry (LC-ESI-MS/MS ) is a recently developed mass spectrometry system with higher sensitivity and reliability compared to MALDI-TOF, making LC-ESI-MS/MS a superior platform for protein identification. The high sensitivity of LC-ESI-MS/MS is achieved through: (1) effective peptide concentration (50–200 fold before MS detection by HPLC column), (2) independent sequencing of peptides, and (3) utilization of between 0.01% and 0.1% of the sample loaded on a MALDI plate during MALDI-based MS analysis, and utilization of almost all the sample during the electrospray process in LC-MS/MS. However, these advanced techniques have not been used to evaluate the pathogenic potential of library books.

In this paper we used a state-of-the-art mass spectrometry instrument, the Thermo™ Fusion™ Tribrid™ Orbitrap mass spectrometer (San Jose, CA, USA), to identify bacteria at the species level. This study aims to identify organisms in children’s books through mass spectrometry and proteomic analysis using an LC-ESI-MS/MS instrument.

## 2. Materials and Methods

### 2.1. Sample Collection

Ten books from each of two public libraries in Houston, Texas, and 11 books from each of two public libraries (a public library in the Central Valley and a public library in the East Bay) in Northern California were swabbed in the winter of 2018. The front and back cover and top and sides of each book were swabbed. Samples were collected using cotton fiber-tipped sterile swabs (Fisher, Cat. No. 14-959-96B). Immediately prior to sample collection, swabs were dipped into the sterile brain–heart infusion (BHI) medium, to ensure bacterial recovery from environmental surfaces [21]. After the book surfaces were sampled, the swab was immediately placed into a 13 mL bacterial culture tube (Sarstedt, 62.515.006), and returned to the laboratory. Separate clean sterile swabs were only dipped into the sterile BHI and placed in 13 mL of bacterial culture before and after sample collection steps per each library for negative control.

### 2.2. Bacterial Cultures, Collection of Bacteria

Cultivation of each sample began by adding 5 mL of BHI medium to the culture tube containing swabs. The tubes were then incubated for 12 h in a 37 °C shake incubator at a speed of 1500 rpm. After initial cultivation, 10 μL of the bacteria culture were inoculated into 5 mL of Luria broth (LB) medium containing 100 μg/mL of ampicillin or 100 μg/mL of kanamycin to select for bacteria grown in ampicillin- or kanamycin-containing medium. After 12 h of shaking incubation the bacteria were pelleted by 2000× *g* spin and PBS-washed.

### 2.3. Proteomics Analysis of Bacteria

The harvested bacteria grown in ampicillin or kanamycin-containing medium were digested and analyzed on LC-ESI-MS/MS based on a previous publication [22]. Briefly, PBS-washed bacteria were suspended with 100 μL of lysis buffer (50 mM ammonium bicarbonate, 1 mM CaCl_2_) and snap-frozen with liquid nitrogen. Then the bacteria were lysed by three cycles of 95 °C boiling and LiN_2_ snap freezing. The protein amount was measured by the Bradford method and 10 μg of total protein were digested with trypsin overnight. The digested peptides were vacuum-dried then dissolved in 30 μL of 5% methanol containing 0.1% formic acid, and one-fifth of the reconstituted samples was subjected to a nLC-1000 (Thermo Fisher Scientific, Waltham, MA, USA) coupled to an Orbitrap Fusion™ Tribrid ™ mass spectrometer (Thermo Fisher Scientific). An in-house trap column (2 cm × 100 μm, Reprosil-Pur Basic C18, 3 μm) was used for enriching peptides. Then, the trap column was switched in-line with an in-house 5 cm × 150 μm capillary column packed with 1.9 um Reprosil-Pur Basic C18 beads. Peptides were separated with a 75-min discontinuous gradient of 4-24% acetonitrile, 0.1% formic acid, at a flow rate of 800 nL/min, then electro-sprayed into a mass spectrometer. The instrument was operated using Xcalibur software ver 4.0 (Thermo Fisher Scientific) in data-dependent mode, acquiring fragmentation spectra of the top 50 strongest ions. Parent MS spectrum was acquired in the Orbitrap with a full MS range of 300–1400 m/z at a resolution of 120,000. HCD fragmented MS/MS spectrum was acquired in an ion-trap in rapid scan mode

### 2.4. Data Analysis with Commercial and In-House Computer Software

Search of the obtained MS/MS spectrum against a target-decoy bacterial ribosome database (48,718 protein sequence entry) was done in the Proteome Discoverer 1.4 interface (ThermoFisher, San Jose, CA, USA) with the Mascot algorithm (Mascot 2.4, Matrix Science, Boston, MA, USA). Oxidation of methionine and protein n-terminal acetylation was allowed as variable modifications. Mass tolerance was 20 ppm for the precursor and 0.5 Dalton for fragment ions. A maximum of two missed cleavages of trypsin digestion was allowed. Assigned peptides were filtered with 1% false discovery rate (FDR). Number of peptide spectrum match (PSM) was used for identification of existing bacteria species.

A Python script was written for wrangling bacterial protein FASTA. A species dependent unique peptide list was created for lysosomal protein to further pinpoint-out identification of specific species level of bacteria strain identification. Briefly, the python script is explained as follows. The SeqIO module available through biopython package was used to read the protein FASTA file. Unique proteins were selected by counting the number of protein accessions where a protein should have only one identifier. Proteins were cleaved to peptide sequence for trypsin using parser module from pyteomics package [23]. Peptides were then mapped to their corresponding protein accession. A list of unique peptides was created where the peptide mapped to only one protein accession. Script available at https://github.com/bbhatt1789/library-germs.git.

### 2.5. Data Availability

The mass spectrometry data have been deposited to the ProteomeXchange Consortium (http:// proteomecentral.proteomexchange.org) via the MASSIVE repository (MSV MSV000083354) with the dataset identifier PXD012473.

## 3. Result

### 3.1. Growth of Bactria in Antibiotic Medium

**Figure 1:**
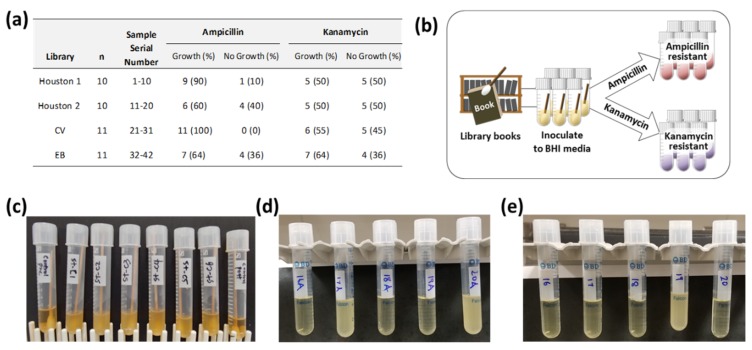
(**a**) summary number of sample origin location, numbers, and sample growth in each antibiotic-containing medium. (**b**) Procedure for antibiotic-resistant bacteria selection. (**c**) Example of culture of collected samples in normal brain–heart infusion (BHI) medium. Two negative controls show no bacteria growth in the first and last position. (**d**) Example of culture BHI growth of bacteria in ampicillin Luria broth (LB) medium. (**e**) Example of culture BHI growth of bacteria in kanamycin LB medium.

In this study, 42 samples were analyzed, with 20 samples referring to Houston libraries (Houston 1 and Houston 2), and 22 samples referring to a public library in the Central Valley (CV) and a public library in the East Bay (EB) in Northern California. Out of 42 samples, all 42 samples were positive in terms of bacterial growth in normal BHI medium (Figure 1c). BHI medium-grown bacterial culture was inoculated into either ampicillin- (Figure 1d) or kanamycin (Figure 1e)-containing LB medium to select antibiotic-resistant bacteria grown in ampicillin or kanamycin-containing medium. After 12 h of incubation in a 37 °C shaking incubator, 33 samples (79%) and 23 samples (55%) were positive in terms of bacterial growth in ampicillin- or kanamycin-containing LB medium, respectively. Culture results in terms of growth of bacteria and libraries identification for samples are shown in Figure 1a. Overall, the occurrence of bacteria grown in ampicillin-containing medium is much higher than the occurrence of bacteria grown in kanamycin-containing medium.

### 3.2. Identified Bacteria Grown in Ampicillin or Kanamycin-Containing Medium

**Figure 2:**
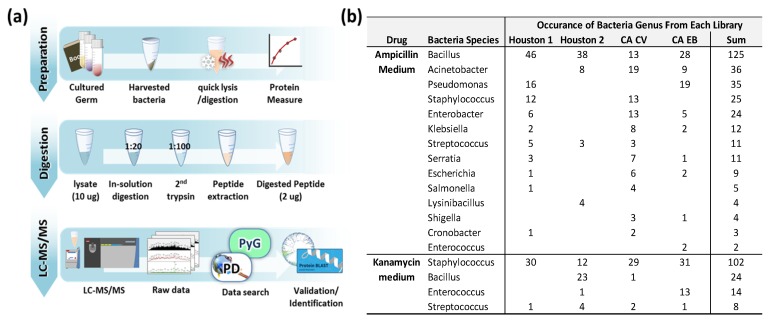
(**a**) Schematic illustration of workflow for identification of bacteria using LC-MS/MS. (**b**) Occurrence of bacteria in genus level from each library depends on different antibiotic medium.

Bacteria grown in ampicillin or kanamycin-containing medium was digested in trypsin and analyzed on LC-ESI-MS/MS as shown in Figure 2a. Acquired MS raw files were searched against bacteria ribosomal FASTA protein database extracted from the NCBI non-redundant RefSeq proteins database (NCBInr) using all known bacteria taxa and ribosome as key words. Because the entire bacteria protein database size was two-thirds of the NCBInr RefSeq database (entry 552,817,090), calculating the raw MS file against it was impractical. Over 12 h would be required to calculate one MS file using the entire bacteria protein database. As a result, we decided to create a far smaller protein database to make data analysis time manageable. Ribosomal proteins are good candidates for such an approach as they are essential proteins regardless of taxa. Although most ribosomal proteins are highly conserved within bacteria, some of these proteins are subject to variations depending on bacteria species so it was proven as a useful tool for the classification of bacterial isolates to the sub-species or strain level [24,25,26,27,28]. The bacteria ribosome protein database containing 48,718 proteins sequence was used reducing the average calculation time for one MS file to 45 min.

Fifteen bacterial genera were discovered from ampicillin-containing culture medium and four bacterial genera were identified from kanamycin-containing culture medium (Figure 2b). The *Bacillus* and *Staphylococcus* genera were the most common genera from ampicillin- and kanamycin-containing media, respectively. The full list of bacteria grown is summarized in Table S1. The identified protein and peptide list for bacteria from ampicillin medium (Table S2) and kanamycin medium (Table S3) is also summarized.

### 3.3. Identified of Bacteria at Species Level

**Figure 3:**
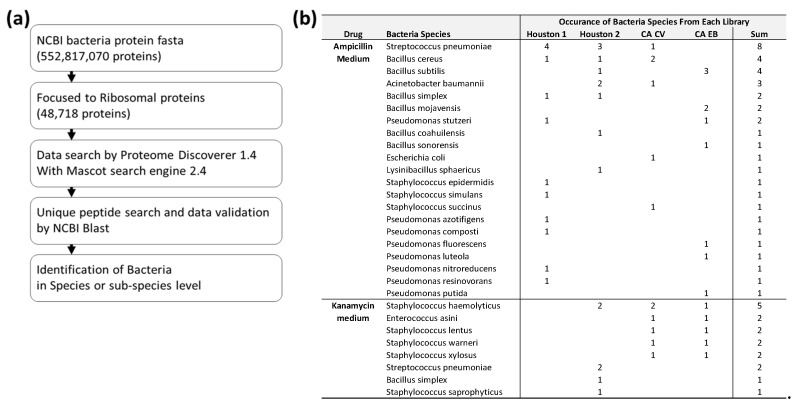
(**a**) Schematic illustration of workflow for identification of bacteria in species or subspecies level using unique peptide identification method. (**b**) Summary of confirmed bacteria species.

We further investigated the recovered peptides from bacteria grown in ampicillin or kanamycin treated media samples to identify bacteria at the species and subspecies level. Determining bacteria at the genus level based on peptides implies some uncertainty since a peptide may be from different genera and species. Therefore, detection of unique peptides of species-specific ribosomal proteins is a promising task for an unambiguous identification of bacteria at the species level. We developed a Python based script that offers the possibility of a highly efficient and simple detection of such unique peptides. The species-specific unique peptide list was generated by the Python script and compared to the recovered peptide list. Any recovered peptide matched to a unique peptide from the Python script indicates the existence of a bacteria species in the sample. Figure 3a shows the work flow of the species-specific peptide identification steps. As shown in Figure 3b, 26 and eight types of bacteria were identified at the species or sub-species level from ampicillin- and kanamycin-containing medium, respectively. The detailed identified bacteria species-specific unique peptide is listed in Table S4. *Streptococcus pneumoniae* was found to be the most common bacteria from ampicillin-containing medium. A few Bacillus species, including *B. cereus* and *B. subtilis* species, were also found as major bacteria (Figure 3b). In kanamycin-containing medium samples, *Staphylococcus haemolyticus* appeared to be most common species, followed by *Enterococcus asini*, as well as a few other *Staphylococcus* species such as *S. lentus*, *S. warneri*, and *S. xylosus*.

## 4. Discussion

Microorganisms exist in every environment where people are active and are generally more beneficial than harmful to humans. The purpose of this study is to investigate the presence of harmful bacteria in children’s books from public libraries by mass spectrometry analysis. Two antibiotics, kanamycin, which mainly works against Gram (-) bacteria, and ampicillin, selective against Gram (+) bacteria, were used to enrich each antibiotic-resistant bacteria from the expected huge numbers of antibiotic-sensitive bacteria on the books. The bacteria grown in kanamycin-containing medium are all Gram (+) bacteria (Figure 2b and Figure 3b).

*S. haemolyticus* is the second most clinically isolated opportunistic pathogen (*S. epidermidis* is the first) that can causes meningitis, skin or soft tissue infections, prosthetic joint infections, or bacteremia [29], and has the highest level of antimicrobial resistance [30]. *S. aureus* is one of the leading pathogens for nosocomial infections showing multi-drug resistance (MDR) [31,32]. All these strains are associated with the human skin, gastrointestinal tract and urogenital tract [33,34].

Both Gram (+) and Gram (-) bacteria are identified in ampicillin containing medium. Gram positive bacteria *S. pneumoniae* was another commonly identified species in this study. *S. pneumoniae* is particularly dangerous for young children, older adults, and persons with underlying comorbidities [35,36]. Gram (-) bacteria *A. baumannii* is known to be one of the most severe MDR pathogens [37]. It often causes problems in immunocompromised individuals, particularly those who have experienced a prolonged (over 90 days) hospital stay [38]. It has been known to spread through the skin as well as the respiratory and oropharyngeal secretions of infected individuals [39]. Because it has an exceptional ability to develop resistance to all currently available antibiotics, it has been designated as a “red-alert” human pathogen [40]. *S. aureus* and *A. baumannii* are members of ESKAPE (acronym of *Enterococcus faecium*, *S. aureus*, *Klebsiella pneumoniae*, *A. baumannii*, *P. aeruginosa*, and *Enterobacter*), which are pathogens commonly associated with MDR [41]. *Bacillus* species are present ubiquitously in nature and are non-pathogenic except two strains: *Bacillus anthracis* which causes anthrax [42], and *B. cereus* which causes food poisoning [43]. Most *Pseudomonas* sp. are naturally resistant to β-lactam antibiotics such as ampicillin [44]; therefore, many *Pseudomonas* species are identified in this study, although the well-known opportunistic human pathogen *P. aeruginosa* species was not identified.

Although some differences are identified depending on the library and area (Texas vs. California), more samples are required to claim any significant differences of the presence of pathogenic bacteria (Figure 2b and Figure 3b).

In addition to the pathogenic bacteria identified in this study, other unidentified pathogenic bacteria could be present in the same sample because pathogenic bacteria were identified on the basis of resistance to ampicillin and kanamycin (Supplemental Table S1).

Species of *Staphylococcus, Bacillus, Enterobacteriaceae*, and *Pseudomonas* are the most common bacteria identified in the hospital environment [3]. All of these species are also identified in children’s books in this study, suggesting that public library books could be responsible for bacterial transmission among children. Our results emphasize the importance of hand sanitizing after reading a book in the library and periodic sterilization of library books.

In this work we applied LC-ESI-MS/MS to detect bacteria at the species level. The advantages of deeper peptide coverage in LC-ESI-MS/MS compared to previously established MALDI-TOF are well addressed in numerous global proteome profiling studies [45,46]. Despite advantages in cost effectiveness and shorter turnaround time, the MALDI-TOF method is only able to detect abundant proteins and identify bacteria down to genus level [20]. For example, Balazova et al. could only identify mycobacterium at the genus level from their study testing the influence of culture conditions using a mixed culture of two known mycobacterium species [47]. Comparatively, we can detect 10,000 peptides within 1 hour of MS instrument time with 10^7^ order of magnitude for protein coverage with our current LC-ESI-MS/MS-based method [22].

## 5. Conclusions

This study describes a simple and rapid method for the direct identification of bacteria from environmental media, which has the potential to discover and identify bacteria from various samples. This technique can be applied to the species or sub-species level of bacteria identification directly from clinical settings such as blood culture. Compared to conventional MALDI-TOF based detection methods, the deeper coverage of the bacterial peptides of this method enables identification of bacteria at the species or sub-species level.

## Supplementary Materials

The following are available online at https://www.mdpi.com/1660-4601/16/6/912/s1, Table S1. List of bacteria from each book samples grown in ampicillin or kanamycin medium. Table S2. Protein and peptide list detected from bacteria grown in ampicillin medium. Table S3. Protein and peptide list detected from bacteria grown in kanamycin medium. Table S4. Bacteria species-specific unique peptide list.

## References

[1] McClary A. (1985). Beware the deadly books: A forgotten episode in library history. J. Libr. Hist..

[2] Brook S.J., Brook I. (1994). Are public library books contaminated by bacteria?. J. Clin. Epidemiol..

[3] Rafiei H., Chadeganipour M., Ojaghi R., Maracy M.R., Nouri R. (2017). The comparison of printed resources bacterial contamination in libraries of Al-Zahra Hospital and Sciences Faculty of Isfahan University and the determination of their antibiotic sensitivity pattern. J. Educ. Health Promot..

[4] Gambale W., Croce J., Costa-Manso E., Croce M., Sales M. (1993). Library fungi at the University of Sao Paulo and their relationship with respiratory allergy. J. Investig. Allergol. Clin. Immunol..

[5] Research Team Seeks Answers on How Dirty the Library Is. https://universe.byu.edu/2011/12/04/dirty-library-books/.

[6] Library Books Could Come with a Side of Germs. https://www.sciencenewsforstudents.org/blog/eureka-lab/library-books-could-come-side-germs.

[7] Bradley J.S., Byington C.L., Shah S.S., Alverson B., Carter E.R., Harrison C., Kaplan S.L., Mace S.E., McCracken G.H., Moore M.R. (2011). The management of community-acquired pneumonia in infants and children older than 3 months of age: clinical practice guidelines by the Pediatric Infectious Diseases Society and the Infectious Diseases Society of America. Clin. Infect. Dis..

[8] Simmonds P.G. (1970). Whole microorganisms studied by pyrolysis-gas chromatography-mass spectrometry: Significance for extraterrestrial life detection experiments. Appl. Microbiol..

[9] Mohamed H., Miloud B., Zohra F., Garcia-Arenzana J.M., Veloso A., Rodriguez-Couto S. (2017). Isolation and Characterization of actinobacteria from Algerian Sahara Soils with antimicrobial activities. Int. J. Mol. Cell Med..

[10] Avanzi I.R., Gracioso L.H., Baltazar M.D., Karolski B., Perpetuo E.A., Do Nascimento C.A. (2017). Rapid bacteria identification from environmental mining samples using MALDI-TOF MS analysis. Environ. Sci. Pollut. Res. Int..

[11] Nacef M., Chevalier M., Chollet S., Drider D., Flahaut C. (2017). MALDI-TOF mass spectrometry for the identification of lactic acid bacteria isolated from a French cheese: The Maroilles. Int. J. Food Microbiol..

[12] Ho Y.P., Reddy P.M. (2010). Identification of pathogens by mass spectrometry. Clin. Chem..

[13] Sauer S., Freiwald A., Maier T., Kube M., Reinhardt R., Kostrzewa M., Geider K. (2008). Classification and identification of bacteria by mass spectrometry and computational analysis. PLoS ONE.

[14] Krasny L., Rohlova E., Ruzickova H., Santrucek J., Hynek R., Hochel I. (2014). Differentiation of Cronobacter spp. by tryptic digestion of the cell suspension followed by MALDI-TOF MS analysis. J. Microbiol. Methods.

[15] He Y., Li H., Lu X., Stratton C.W., Tang Y.W. (2010). Mass spectrometry biotyper system identifies enteric bacterial pathogens directly from colonies grown on selective stool culture media. J. Clin. Microbiol..

[16] Richter S.S., Sercia L., Branda J.A., Burnham C.A., Bythrow M., Ferraro M.J., Garner O.B., Ginocchio C.C., Jennemann R., Lewinski M.A. (2013). Identification of Enterobacteriaceae by matrix-assisted laser desorption/ionization time-of-flight mass spectrometry using the VITEK MS system. Eur. J. Clin. Microbiol. Infect. Dis..

[17] Clark C.G., Kruczkiewicz P., Guan C., McCorrister S.J., Chong P., Wylie J., Van Caeseele P., Tabor H.A., Snarr P., Gilmour M.W. (2013). Evaluation of MALDI-TOF mass spectroscopy methods for determination of Escherichia coli pathotypes. J. Microbiol. Methods.

[18] Angeletti S., Dicuonzo G., D’Agostino A., Avola A., Crea F., Palazzo C., Dedej E., De Florio L. (2015). Turnaround time of positive blood cultures after the introduction of matrix-assisted laser desorption-ionization time-of-flight mass spectrometry. New Microbiol..

[19] Curtoni A., Cipriani R., Marra E.S., Barbui A.M., Cavallo R., Costa C. (2017). Rapid identification of microorganisms from positive blood culture by MALDI-TOF MS after short-term incubation on solid medium. Curr. Microbiol..

[20] Sloan A., Wang G., Cheng K. (2017). Traditional approaches versus mass spectrometry in bacterial identification and typing. Clin. Chim. Acta.

[21] Afshinnekoo E., Meydan C., Chowdhury S., Jaroudi D., Boyer C., Bernstein N., Maritz J.M., Reeves D., Gandara J., Chhangawala S. (2015). Geospatial resolution of human and bacterial diversity with city-scale metagenomics. Cell Syst..

[22] Jung S.Y., Choi J.M., Rousseaux M.W., Malovannaya A., Kim J.J., Kutzera J., Wang Y., Huang Y., Zhu W., Maity S. (2017). An anatomically resolved mouse brain proteome reveals parkinson disease-relevant pathways. Mol. Cell Proteom..

[23] Goloborodko A.A., Levitsky L.I., Ivanov M.V., Gorshkov M.V. (2013). Pyteomics–A Python framework for exploratory data analysis and rapid software prototyping in proteomics. J. Am. Soc. Mass Spectrom..

[24] Suarez S., Ferroni A., Lotz A., Jolley K.A., Guérin P., Leto J., Dauphin B., Jamet A., Maiden M.C., Nassif X. (2013). Ribosomal proteins as biomarkers for bacterial identification by mass spectrometry in the clinical microbiology laboratory. J. Microbiol. Methods.

[25] Pineda F.J., Antoine M.D., Demirev P.A., Feldman A.B., Jackman J., Longenecker M., Lin J.S. (2003). Microorganism identification by matrix-assisted laser/desorption ionization mass spectrometry and model-derived ribosomal protein biomarkers. Anal. Chem..

[26] Ryzhov V., Fenselau C. (2001). Characterization of the protein subset desorbed by MALDI from whole bacterial cells. Anal. Chem..

[27] Teramoto K., Sato H., Sun L., Torimura M., Tao H., Yoshikawa H., Hotta Y., Hosoda A., Tamura H. (2007). Phylogenetic classification of Pseudomonas putida strains by MALDI-MS using ribosomal subunit proteins as biomarkers. Anal. Chem..

[28] Teramoto K., Sato H., Sun L., Torimura M., Tao H. (2007). A simple intact protein analysis by MALDI-MS for characterization of ribosomal proteins of two genome-sequenced lactic acid bacteria and verification of their amino acid sequences. J. Proteome. Res..

[29] Falcone M., Campanile F., Giannella M., Borbone S., Stefani S., Venditti M. (2007). Staphylococcus haemolyticus endocarditis: Clinical and microbiologic analysis of 4 cases. Diagn. Microbiol. Infect. Dis..

[30] Barros E.M., Ceotto H., Bastos M.C., Dos Santos K.R., Giambiagi-Demarval M. (2012). Staphylococcus haemolyticus as an important hospital pathogen and carrier of methicillin resistance genes. J. Clin. Microbiol..

[31] Tong S.Y., Davis J.S., Eichenberger E., Holland T.L., Fowler V.G. (2015). Staphylococcus aureus infections: epidemiology, pathophysiology, clinical manifestations, and management. Clin. Microbiol. Rev..

[32] Lister J.L., Horswill A.R. (2014). Staphylococcus aureus biofilms: recent developments in biofilm dispersal. Front Cell Infect. Microbiol..

[33] Human Microbiome Project Consortium (2012). A framework for human microbiome research. Nature.

[34] Human Microbiome Project Consortium (2012). Structure, function and diversity of the healthy human microbiome. Nature.

[35] Ortqvist A., Hedlund J., Kalin M. (2005). Streptococcus pneumoniae: Epidemiology, risk factors, and clinical features. Semin. Respir. Crit Care Med..

[36] Torres A., Blasi F., Dartois N., Akova M. (2015). Which individuals are at increased risk of pneumococcal disease and why? Impact of COPD, asthma, smoking, diabetes, and/or chronic heart disease on community-acquired pneumonia and invasive pneumococcal disease. Thorax.

[37] Rice L.B. (2008). Federal funding for the study of antimicrobial resistance in nosocomial pathogens: No ESKAPE. J. Infect. Dis..

[38] Montefour K., Frieden J., Hurst S., Helmich C., Headley D., Martin M., Boyle D.A. (2008). Acinetobacter baumannii: An emerging multidrug-resistant pathogen in critical care. Crit Care Nurse.

[39] Sebeny P.J., Riddle M.S., Petersen K. (2008). Acinetobacter baumannii skin and soft-tissue infection associated with war trauma. Clin. Infect. Dis..

[40] Cerqueira G.M., Peleg A.Y. (2011). Insights into Acinetobacter baumannii pathogenicity. IUBMB Life.

[41] Santajit S., Indrawattana N. (2016). Mechanisms of antimicrobial resistance in ESKAPE pathogens. Biomed. Res. Int..

[42] Spencer R.C. (2003). Bacillus anthracis. J. Clin. Pathol..

[43] Kotiranta A., Lounatmaa K., Haapasalo M. (2000). Epidemiology and pathogenesis of Bacillus cereus infections. Microbes Infect..

[44] Palleroni N.J. (2010). The Pseudomonas story. Environ. Microbiol..

[45] Zubarev R.A., Makarov A. (2013). Orbitrap mass spectrometry. Anal. Chem..

[46] Hessling B., Buttner K., Hecker M., Becher D. (2013). Global relative quantification with liquid chromatography-matrix-assisted laser desorption ionization time-of-flight (LC-MALDI-TOF)—Cross-validation with LTQ-Orbitrap proves reliability and reveals complementary ionization preferences. Mol. Cell Proteom..

[47] Balazova T., Makovcova J., Sedo O., Slany M., Faldyna M., Zdrahal Z. (2014). The influence of culture conditions on the identification of Mycobacterium species by MALDI-TOF MS profiling. FEMS Microbiol. Lett..

